# Giant Cell Reparative Granuloma Mimicking Aneurysmal Bone Cyst in Proximal Phalanx of Toe

**DOI:** 10.5704/MOJ.1603.011

**Published:** 2016-03

**Authors:** CM Huan, AB Norzila

**Affiliations:** Department of Orthopaedics, Hospital Universiti Sains Malaysia, Kubang Kerian, Kelantan Malaysia

**Keywords:** Giant Cell Reparative Granuloma, Aneurysmal Bone Cyst

## Abstract

Giant Cell Reparative Granuloma (GCRG) of phalanx is uncommon. It is a benign osteolytic lesion but can be locally aggressive. GCRG has certain radiology and histological features that are similar to other giant cell lesions of the bone. We present a case report of a young patient with giant cell reparative granuloma of proximal phalanx of left third toe. The bone lesion was successfully treated surgically.

## Introduction

Giant Cell Reparative Granuloma (GCRG) is a benign osteolytic bone lesion. It rarely affects the tubular bones of the hand and foot. In 1953, Jaffe first described GCRG which was found in a patient with mandible and maxilla lesion^1^ and subsequently described similar findings in the hand and foot by other authors. GCRG has similar characteristics with other giant cell lesions especially giant cell tumor, aneurismal bone cyst and brown tumor. Thus, our report emphasizes on radioimaging and hispathological findings in this patient.

## Case Report

**Figure 1(a), (b):**
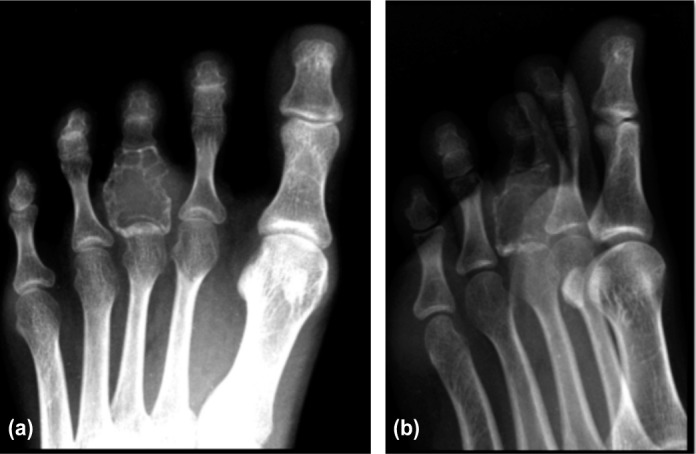
Left foot radiograph shows expansile lytic bone lesion in the proximal phalanx of left third toe. Multiple septations can be seen within the lesion. The cortical margin of the bone remains intact.

**Figure 2(a), (b):**
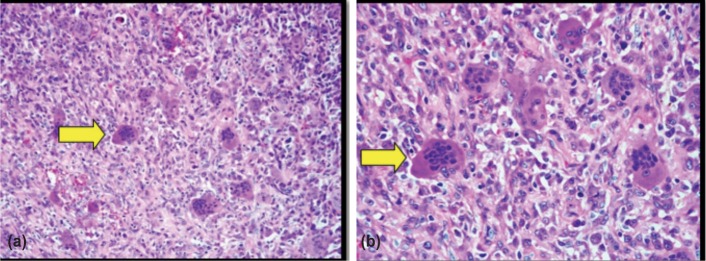
High-power photomicrograph (H-E stain) (a) 20x and (b) 40x show both multinucleated giant cells (yellow arrows) the intervening mononuclear cells and spindle shape fibroblasts.

A 21 year-old Malay lady came to Orthopedic clinic, complaining of 2 years duration of left third toe swelling. The swelling was progressively increased in size and was associated with occasional pain for the past 1 year. There was no history of trauma or infection prior to the toe swelling. Hematological investigations were unremarkable. Diagnosis of aneurismal bone cyst was made based on radiograph findings (Fig 1a,b). Patient underwent surgical excision of the bone lesion with bone grafting from iliac bone and K wire insertion. Histopathological examination (HPE) showed giant cell reparative granuloma (Fig 2a,b).

## Discussions

The clinical presentations of GCRG in the distal phalanx are non specific. Typically patient presents with pain, swelling, tenderness and palpable mass at the phalanx. The duration of symptoms ranges from 6 weeks to 10 years^2^. Our patient presented with 2 year duration of relative painless lesion at the left third toe.

Radiologically GCRG is difficult to distinguish from Aneurysmal bone cyst (ABC). Both lesions show expansile lytic lesion with multilocular appearance and thin cortex^3^. Although MRI typically shows fluid-fluid levels within the cystic cavities of ABC, this feature is not always present. HPE was previously used to differentiate GCRG from ABC. However in recent years, a study on ABC has found similar morphology to GCRG. A term of ‘solid variant’ was added to ABC^4^. The solid variant of aneurysmal bone cyst and GCRG were later described as reactive and benign.

**Figure 3(a), (b):**
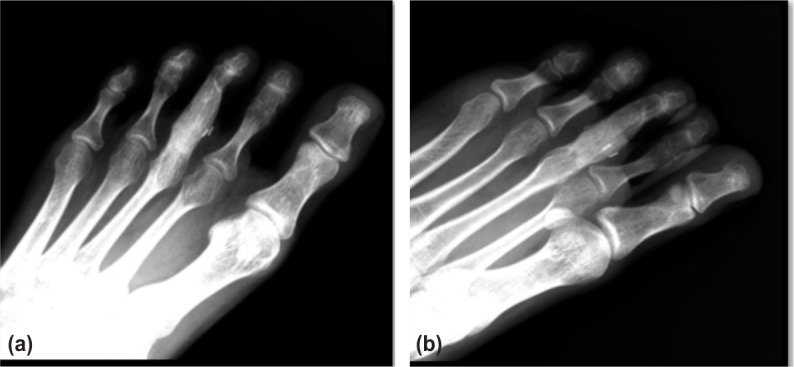
Left foot radiograph 8 months post surgery shows good healing process with no local recurrence.

Conventional treatment of GCRG is enucleation and curettage. The recurrence rate of this method is estimated between 10% to 20%^5^. Complete surgical resection of the lesion reduces the recurrence rate but at the expense of lost of function. Our patient underwent surgical wide resection of bone lesion with iliac bone grafting. She recovered well after surgery. Follow up radiograph showed no recurrence of the lesion after 8 months of surgery (Figure 3). She was able to ambulate well and having normal daily lives.

## Conclusion

GCRG can mimic ABC in plain radiograph. Although GCRG is rare especially in the distal phalanx, physicians should be aware of various differential diagnoses when dealing with expansile lytic bone lesion in the distal phalanx.

## References

[1] Jaffe HL (1953). Giant-cell reparative granuloma, traumatic bone cyst, and fibrous (fibro-osseous) dysplasia of the jawbones. Oral Surg, Oral Med, Oral Pathol.

[2] Giza E, Stern PJ, Cualing H (1997). Aggressive giant cell reparative granuloma of the metacarpal: a case report. J Hand Surg Am.

[3] Murphey MD, Nomikos GC, Flemming DJ (2001). Imaging of Giant Cell Tumor and Giant Cell Reparative Granuloma of Bone: Radiologic-Pathologic Correlation. Radiographics.

[4] Sanerkin NG, Mott MG, Roylance J (1983). An unusual intraosseous lesion with fibroblastic, osteoclastic, osteoblastic, aneurysmal and fibromyxoid elements. “Solid” variant of aneurysmal bone cyst. Cancer.

[5] Pogrel AM (2012). The diagnosis and management of giant cell lesions of the jaws. Ann Maxillofac Surg.

